# Primary and secondary anti-tuberculosis drug resistance in Hitossa District of Arsi Zone, Oromia Regional State, Central Ethiopia

**DOI:** 10.1186/s12889-016-3210-y

**Published:** 2016-07-18

**Authors:** Shallo Daba Hamusse, Dejene Teshome, Mohammed Suaudi Hussen, Meaza Demissie, Bernt Lindtjørn

**Affiliations:** Oromia Regional Health Bureau, Addis Ababa, Ethiopia; Adama Regional Research Center Laboratory, Adama, Ethiopia; Addis Continental Institute of Public Health, Addis Ababa, Ethiopia; Centre for International Health, University of Bergen, Bergen, Norway

**Keywords:** Primary and secondary MDR-TB, Hitossa District, Ethiopia

## Abstract

**Background:**

Multidrug-resistant tuberculosis (MDR-TB) drugs which is resistant to the major first-line anti-TB drugs, Isoniazid and Rifampicin, has become a major global challenge in tuberculosis (TB) control programme. However, its burden at community level is not well known. Thus, the aim of study was to assess the prevalence of primary and secondary resistance to any first line anti-TB drugs and MDR TB in Hitossa District of Oromia Regional State, Central Ethiopia.

**Methods:**

Population based cross- sectional study was conducted on individuals aged ≥15 years. Those with symptoms suggestive of TB were interviewed and two sputum specimens were collected from each and examined using Lowenstein-Jensen (LJ) culture medium. Further, the isolates were confirmed by the Ziehl-Neelsen microscopic examination method. Drug susceptibility test (DST) was also conducted on LJ medium using a simplified indirect proportion method. The resistance strains were then determined by percentage of colonies that grew on the critical concentration of Isoniazid, Streptomycin, Rifampicin and Ethambutol.

**Results:**

The overall resistance of all forms of TB to any first-line anti-TB drug was 21.7 %. Of the total new and previously treated culture positive TB cases, 15.3 and 48.8 % respectively were found to be a resistant to any of the first-line anti-TB drugs. Further, of all forms of TB, the overall resistance of MDR-TB was 4.7 %. However, of the total new TB cases, 2.4 % had primary while 14.3 % had secondary MDR-TB. Resistance to any of the first-line anti-TB drugs (adjusted odd ratio (AOR), 8.1; 95 % CI: 2.26–29.30) and MDR-TB (AOR), 7.1; 95 % CI: 2.6–43.8) was found to be linked with previous history of anti-TB treatment.

**Conclusions:**

The study has identified a high rate of primary and secondary resistance to any of the first-line anti-TB drugs and MDR-TB in the study area. The resistance may have resulted from sub-optimal performance of directly observed treatment short-course (DOTS) programme in the detecting infectious TB cases and cure rates in the study area. Anti-TB drug resistance is linked with previous TB treatment. There is a need to strengthen DOTS and DOTS-Plus programmes and expand MDR-TB diagnostic facilities in order to timely diagnose MDR-TB cases and provide appropriate treatment to prevent the spread of MDR-TB in Ethiopia.

**Electronic supplementary material:**

The online version of this article (doi:10.1186/s12889-016-3210-y) contains supplementary material, which is available to authorized users.

## Background

Tuberculosis (TB) remains a high-priority communicable disease that causes illness among millions of people and is the second leading cause of death from an infectious disease worldwide [1]. The disease disproportionately affects people in resource-poor settings, particularly those in Asia and Africa. More than 80 % of TB cases and 78 % of deaths from the disease occurred in developing countries [2, 3]. In 2013, there were 9.0 million TB new cases, and 1.5 million TB deaths occurred around the globe [2]. Ethiopia has been listed as one of the 22 high-TB burden countries with respective mortality, prevalence and incidence rate of 32, 211 and 224 cases per 100,000 population. Moreover, 11 % of patients with TB are also infected by the Human Immunodeficiency Virus (HIV) [2].

Multidrug-resistant tuberculosis (MDR-TB) is defined as Mycobacterium tuberculosis strain resistant to at least the first-line anti-TB drugs of Isoniazid and Rifampicin. [1]. MDR-TB occurs either when a person is infected with a resistant strain or when insufficient or improper treatment leads to drug selection of the resistant strain [1]. When a person with no history of first-line anti-TB treatment develops MDR-TB, it is known as primary resistance to any first line anti- TB drugs and MDR-TB, whereas when a person with a history of first-line anti-TB treatment acquires resistance to any first line anti-TB drugs and MDR-TB, they are respectively called secondary resistance to any first line anti-TB drugs and MDR-TB [4].

Multidrug-resistant TB has been known to be a major challenge in TB control programme. It has been spreading rapidly across the globe, and in recent years an estimated 3.5 % of new cases and 20.5 % of previously treated TB cases have MDR-TB. In 2013, there were an estimated 480,000 MDR-TB cases, and about 210,000 deaths were caused by MDR-TB worldwide [2]. The prevalence of one or more drug-resistant TB and MDR-TB varies across counties with more than half of MDR-TB cases occurring in India, China and the Russian Federation [2]. The most difficult and complicated form of drug resistant TB is known as extensively drug resistant tuberculosis (XDR-TB); this has been reported from 92 countries (eight from Africa including Ethiopia) [5]. Globally 9 % of MDR-TB cases have XDR-TB [2, 6].

According to the 2014 World Health Organization (WHO) report, Ethiopia ranked 15^th^ out of the 27 countries with the highest estimated number of multidrug resistant *tuberculosis* (MDR-TB) cases. Additionally, WHO estimated the prevalence of MDR-TB among new TB cases in Ethiopia at 1.6 % (95 % CI, 0.9 to 2.8 %), and among the previously treated ones at 12 % (95 % CI: 5.6 to 21 %) [2]. Furthermore, according to the 2014 National Anti-tuberculosis Drug Resistance Survey, the prevalence of MDR-TB among new, previously treated and overall TB cases were 2.3, 17.8 and 4.8 %, respectively [7]. Moreover, study reports from Eastern, Central, Northern and Southern Ethiopia have shown that the prevalence of primary resistance MDR-TB ranged from 1.1 to 5.8 % [8–11] and secondary resistance MDR-TB from 10.9 to 71.4 % [9, 10, 12, 13].

Nevertheless, to our knowledge, reports from Ethiopia on the prevalence of MDR-TB including primary and secondary resistance were based on reports among health service seekers from health facilities and might therefore be subject to selection bias [14–16].

Because of the absence of population-based studies and adequate laboratory testing facilities in the country, it is difficult to have a reliable estimation on the burden of primary and secondary resistance to any first line anti-TB drugs and MDR TB. However, this population-based study conducted in Hitossa District of Arsi zone Oromia Regional State, Central Ethiopia was the first attempt in the country aimed at measuring the prevalence of primary and secondary resistance to any first line anti-TB drugs and MDR-TB.

## Methods

### Study setting and population

The study was conducted in Hitossa District of Arsi Zone, Oromia Regional State in Central Ethiopia. The population density, socio–economic state, and demographic condition of Hitossa are typical representative of the entire features of Arsi Zone. The Directly Observed Treatment, Short-Course (DOTS) and health service coverage, the proportion of urban–rural population and HIV prevalence, and the TB case notification and treatment outcomes of Hitossa District are also similar to those of the whole zone of Arsi. The district has an estimated population of 178,229 people living in 23 rural and one urban *kebele* (the smallest administrative unit in government structure) [17]. Since 2010, *kebeles* have been further divided into three sub-*kebeles* known as *gote*. Each *gote* has about 1/3 population of the *kebele* [17].

### Study design

Population based cross sectional study was conducted between 1 July 2013 and 30 June 2014 to estimate the prevalence of primary and secondary multi-drug resistant to any first line anti-TB drugs and MDR-TB in Hitossa District of Arsi Zone. For practical and economic reasons Hitossa District was selected purposefully and all its *kebeles* were included in the study. One *gote* from each *kebele* was randomly selected, and individuals 15 years and above living in the households of the selected *gote* were enrolled in the study. A week before the baseline survey, pre-survey registration was carried out in the selected *gote*s of the 24 *kebeles*. Accordingly, 61,678 individuals living in 9,454 households were identified. Eligibility includes permanent residents of 15 years of age and above who could provide written consent for willingness to participate in the study and temporary visitors who arrived at least 15 days before the commencement of the study. Using the above criteria, 33,073 adults were identified and included in the study.

### Data collection procedures

The aims of the study and the procedures of data collection were discussed with zonal, district and *kebele* leaders. A total of 24 teams with one nurse and one health extension worker (HEW) were involved in the data collection. Furthermore, five laboratory technicians also took part in sputum sample collection. The whole process of data collection was supervised by 10 health officers. All laboratory technicians and data collectors were selected from public health institutions in the study district, and they were trained on TB screening techniques and on how to collect and transport sputum specimen. The same team of data collectors was assigned to each *kebele* for both pre-survey registration and data collection. The survey was carried out three times at interval of 6 months: the first at baseline, the second at the end of the sixth month, and the third at the end of the 12th month between 1 July 2013 and 30 June 2014.

House to house visits were carried out to identify individuals with persistent cough of more than two weeks, fever, and loss of appetite, weight loss, blood-stained sputum and chest pain or difficulty of breathing which were considered as symptoms suggestive of pulmonary TB. Individuals with such symptoms were interviewed on their socio–economic and demographic information and current and previous history of TB treatment. Subsequently, participants with any symptom suggestive of the illness were asked to submit two adequate recently-discharged mucoid or muco-purulent sputum specimens (spot-morning). The laboratory technicians collected the sputum in sterile falcon tubes and immediately placed them in a cold box at 4 °C, and transported them on the same day to Adama Regional Research Center Laboratory.

### Culture and identification

On the following day of the sputum receipt, the morning specimen was digested and decontaminated by the standard Acetyl L-cysteine (NALC)-NaOH method [18], followed by centrifuged at 800 X g for 15 min to concentrate the organisms. The sediment (pellet) was reconstituted with 2.5 ml of sterile phosphate buffer (pH 6.8) to prepare the suspensions for the cultures. The sediments were inoculated in to egg-based Löwenstein-Jensen (LJ) medium slant tubes prepared based on the International Union against Tuberculosis and Gung Disease (IUATLD) for the primary isolation of the organisms [19]. Following that the LJ slant tubes were incubated at 37 °C and inspected for a period of eight weeks for the growth of *Mycobacterium tuberculosis* complex. The cultures were considered to be negative if no colonies were identified after 8 weeks of incubation. Moreover, the isolates from the LJ were confirmed by microscopic examination for the presence of Acid-Fast Bacillus (AFB) using Ziehl-Neelsen method. *Moreover*, susceptibility test of all isolates to spara-nitrobenzoic acid was carried out to identify *Mycobacterium tuberculosis complex* from environmental mycobacteria. However, all isolates were found to be *Mycobacterium tuberculosis complex* [19].

### Drug susceptibility test

Drug susceptibility tests(DST) were carried out using the simplified indirect proportion method on LJ medium [19]. The proportion method validates the percentage of growth of distinct inoculums on a drug-free control medium compared to growth on culture media containing the critical concentration of anti-tuberculosis drugs. The resistant strains were determined using the percentage of colonies that grew on the critical concentration of 0.2 mg/l for Isoniazid, (INH), and 4 mg/l for Streptomycin (STM), 40 mg/l for Rifampicin (RIF) and 2 mg/l for Ethambutol (EMB). The isolate was said to be drug resistant when the growth was more than or equal to 1 % of the bacterial population on the media containing the critical concentration of each drug [19].

### Data management and analysis

To ensure data quality, the principal investigator and supervisors closely monitored the data collection process in which a standardized and pre-tested questionnaire was used. The collected data were coded and double entered into Epi-info version 7 statistical software by trained data clerks. Then they were checked against the original information for missing variables. Errors were corrected by referring to the original questionnaire.

The percentage of drug resistance to any of the drugs or its combination with other drugs was determined. The primary resistance was calculated by dividing the number of resistant isolates among new TB cases by the total number of new TB cases found to be culture positive for a particular drug or combination of drugs multiplied by 100. Similarly, percentage of secondary resistance was calculated by dividing the number of resistant isolates in previously treated TB cases by the total number of re-treated TB cases found to be culture positive for that particular drug or combination of drugs multiplied by 100. We excluded 20 contaminated cultures from the analysis as their results were not known.

Analysis was made using IBM SPSS version 20 statistical software (SPSS Inc. Chicago. 2007). Descriptive analysis was made and frequencies and odds ratios (OR) with the 95 % confidence intervals (CI) were calculated. Logistic regression analysis was used to evaluate the association between drug resistance as outcome and others related independent variables. A significance level of <0.05 was considered statistically significant. We also compare the result of current study with those of other studies carried out in Ethiopia between 1984 and 2015 to understand the trend of drug resistance over time (Table 4).

## Results

### General characteristics of the study population

A total of 61,678 individuals in 9,454 households from the study area were identified. Of these, 33,073 were found to be eligible, and screened for symptoms suggestive of PTB. Of these, 16,888 (51 %) were males and 28,048 (84.8 %) were rural residents. The age of the respondents ranged from 15 to 94 years, with a mean age of 42.3 (±18.4 SD) years. Out of the eligible individuals, 2,758 (8.3 %) reported to have symptoms suggestive of PTB. Among these, 1,717 (62.3 %) were females and 1041 (37.7 %) were males. Of the 2,758 suspected cases, 2, 218 (80.4 %) were new, and 540 (19.6 %) had previously been treated with first-line anti-TB drugs (Table 1).

**Table 1:** General characteristics of TB suspects, smear culture positive and MDR-TB cases in Hitossa District, Arsi Zone of Oromia Region, Central Ethiopia, 2015

Characteristics	TB suspects (*N* = 2,758)	Culture positive TB cases (*N* = 106)
Sex	n (%)	n (%)
Male	1041 (37.7)	51 (48.1)
Female	1717 (62.3)	55 (51.9)
Total	2758 (100)	106 (100)
Age		
15–24	543 (19.7)	40 (37.7)
25–34	525 (19.0)	18 (17.0)
35–44	448 (16.3)	29 (27.4)
≥ 45	1242 (45.0)	19 (17.9)
Total	2758 (100)	106 (100)
Residence		
Rural	2277 (82.6)	58 (54.7)
Urban	481 (17.4)	48 (45.3)
Total	2758 (100)	106 (100)
Education		
Literate	1212 (43.9)	47 (44.3)
Illiterate	1546 (56.1)	59 (55.7)
Total	2758 (100)	106 (100)
History of pervious TB treatment		
No	2218 (80.4)	85 (80.2)
Yes	540 (19.6)	21 (19.8)
Total	2758 (100)	106 (100)

### Drug susceptibility pattern

All the 2,758 TB suspect cases gave spot and morning sputum for culture examination. Of these, 106 (3.8 %) were found to be culture positive for *Mycobacterium tuberculosis*. Twenty specimens were contaminated and were therefore excluded from the analysis. From the total 106 culture positives, 85 (80.2 %) were new, whereas 21 (19.8 %) were previously treated patients with first-line anti-TB drugs (Table 1). Of the 106 isolates, 83 (78.3 %) were susceptible to all first-line anti-TB drugs (Streptomycin (STM), Isoniazid (INH), Ethambutol (EMB) and Rifampicin (RIF)), while 23 (21.7 %) were resistant to one or to a combination of the first-line anti-TB drugs. Five cultures (4.7 %, 95 % CI: 2.8–6.6 %) were MDR-TB cases.

Of the 85 new *M. tuberculosis* isolates, primary resistant strains to any first-line anti-TB drugs were observed in 13 (15.3 %) patients. Of these, 8 (9.4 %) were resistant to each INH and STM, 4 (4.7 %) to RIF and 3 (3.5 %) to EMB. Primary MDR-TB was detected in 2 (2.4 %) strains (Table 3). However, among the new cases, no isolates of primary resistance to all first-line drugs were observed. Primary mono-resistance to each of INH and STM was found in two cultures (2.4 %). There was no primary mono-resistance to RIF and EMB among the new isolates (Table 2).

**Table 2:** Primary and secondary drug resistance pattern to first-line anti-TB drugs among culture positive pulmonary TB cases in Hitossa District, Arsi Zone of Oromia Region, Central Ethiopia 2015

Drug resistance pattern	New cases (*N* = 85)	Re-treated cases (N-21)	Total (*N* = 106)
	n (%) (95 % CI)	n (%) (95 % CI)	n (%) 95 % CI
Any R to one drug	13 (15.2 % (7.6–22.8)	11 (52.3: 30.9–73.7)	24 (22.6: 14.6–30.6)
Any INH	8 (9.4 %)	6 (28.6)	14 (13.2)
Any RIF	4 (4.7)	4 (19.0)	8 (7.5)
Any STM	8 (9.4)	5 (23.8)	13 (12.3)
Any EMB	3 (3.5)	2 (9.5)	5 (4.7)
Mono resistance	4	4	8
Only INH	2 (2.4)	1 (4.8)	3 (2.8)
Only RIF	0	0	0
Only STM	2 (2.4)	2 (9.5)	4 (3.8)
Only EMB	0	1 (4.8)	1
Two-drug resistance	7	4	11
INH + RIF	1 (1.2)*	1 (4.8)	2 (1.9)*
INH + ETM	1 (1.2)	0	1 (0.9)
INH + STM	2 (2.4)	2 (9.5)	4 (3.8)
RIF + EMB	0	0	0
RIF + STM	2 (2.4)	1 (4.8)	3 (2.8)
ETM + STM	1 (1.2)	0	1 (0.9)
Three or more-drug resistance	2	3	5
INH + RIF + EMB	0	0	0
INH + RIF + STM	1 (1.2)*	2 (9.5)	3 (2.8)*
INH + EMB + STM	1 (1.2)	0	1 (0.9)
RIF + ETM + STM	0	0	0
INH + RIF + EMB + STM	0	1 (5.9)	1 (0.9)
MDR*	2 (2.4)	3 (14.3)	5 (4.7)

NB: MDR-TB* is multi-drug resistant TB

Moreover, out of the 21 previously treated *M. tuberculosis* isolates, strains of secondary resistance to any of the first-line anti-TB drugs were identified in 11 (52.2 %) patients. Of these, 6 (28.6 %), were resistant to INH, 5 (23.8 %) to STM, and 4 (19 %) to RIF. Secondary MDR-TB was detected in 3 (14.3 %) isolates. Of these, one (4.8 %) was found to be resistant to all first-line drugs (Table 3). Among previously treated cases, the highest mono-resistance was to STM with 2 (9.5 %), followed by one (4.8 %) to each INH and EMB. However, among the previously treated TB cases, no mono-resistance to RIF was reported (Table 2).

**Table 3:** Pattern of drug resistance among culture-positive TB cases with different variables in Hitossa District, Arsi Zone of Oromia Region, Central Ethiopia, 2015

Characteristics	Culture positive (*n* = 106)	MDR-TB cases (*n* = 5)			Any TB drug resistance (*n* = 23)		
		Number	COR (95 % CI)	AOR (95 % CI)	Number	COR (95 % CI)	AOR (95 % CI)
Sex							
Male	51 (48.1)	2	0.71 (0.11–4.4)	0.67 (0.10–4.2)	14	1.54 (0.61–3.90)	2.5 (0.78–8.05)
Female	55 (51.9)	3	1.00	1.00	10	1.00	1:00
Total	106 (100)	5			24		
Age							
15–24	40 (37.7)	3	1:00	1.00	10	1.00	1:00
25–34	18 (17.0)	0	-------	------	6	1.5 (0.45–5.10)	2:18 (0.55–8.70)
35–44	29 (27.4)	2	0.91 (0.14–5.84)	0.90 (0.3–6.1)	6	0.63 (0.19–2.10)	0.36 (0.08–1.56)
≥ 45	19 (17.9)	0	-------	-------	2	0.35 (0.07–1.80)	0.19 (0.03–1.35)
Total	106 (100)	5			24		
Residence							
Rural	58 (54.7)	1	1:00	1.00	9	1.00	1:00
Urban	48 (45.3)	4	5.18 (0.56–4.8)	4.8 (0.7–4.7)	15	2.84 (1.11–7.45)	4.1 (1.33–12.84)
Total	106 (100)	5			24		
Education							
Literate	47 (44.3)	3	1:00	1.00	10	1.00	1:00
Illiterate	59 (55.7)	2	0.51 (0.08–3.21)	0.63 (0.09–3.6)	14	1.31 (0.51–3.37)	1.64 (0.53–5.11)
Total	106 (100)	5			24		
History of TB treatment							
No	85 (80.2)	2	1:00	1.00	14	1.00	1:00
Yes	21 (19.8)	3	6.92 (1.10–44.4)	7.1 (2.6–43.8)	10	3.48 (1.29–9.44)	8:13 (2.26–29.30)
Total	106 (100)	5			24		

*COR* Crude Odds Ratio and *AOR* Adjusted Odds Ratio

### Risk factors associated with drug resistance

Previous history of TB treatment, and urban residence were independently associated with high risk of resistance to any first-line anti-TB drug. Individuals with pervious history of TB treatment were eight times (adjusted odd ratio (AOR), 8.1; 95 % CI: 2.3–29.3) more likely to develop resistance to any first-line anti-TB drugs compared to those with no history of previous TB treatment. Similarly, urban residents were four times (AOR, 4.1; 95 % CI: 1.3–12.8) more likely to have resistance to any first-line anti-TB drugs compared to their rural counterparts. Individuals who had pervious history of TB treatment were nearly seven times more likely (AOR 7.1; 95 % CI: 2.6–43.8)) to have MDR-TB compared to those who had no history of pervious exposure to anti-TB drugs (Table 3).

### Rates of anti-TB drug resistance between 1984 and 2015

A total of 18 studies that had been published between 1994 and 2015 on the primary and secondary anti-TB drug resistance were reviewed. Results showed that primary resistance to any drug raged from 10.7 % in 2009 to 30.1 % in 2012. Moreover, the rate of primary MDR-TB increased from 0.6 in 1994 to 3.7 % in 2009 while that of secondary resistance to any anti-TB drugs varied from 11.1 % in 1996/7 to 85.7 % in 2005/6. According to reports from the same studies, the rate of secondary MDR-TB ranged from 15.7 % in 1996/7 and 60.8 % in 2005/6 (Table 4).

**Table 4:** Rate of anti-TB drug resistance in current and other studies conducted between 1984 and 2015 in Ethiopia

Type of the study		Study time	Sample size	Rate of anti-TB resistance					
				Primary resistance		Secondary resistance		Overall resistance (primary plus secondary resistance)	
				Any-resistance (%)	MDR-TB (%)	Any- resistance (%)	MDR-TB (%)	Any- resistance (%)	MDR-TB (%)
Community-based	Institution-based								
Current study		2014	33073^a^	15.3	2.4	48.8	14.3	21.7	4.7
	Demissie M et al. [34]	1994	167^b^	15.6	0.6				
	Bruchfeld et. al [22]	1996–1997	509	14.6	0.9	11.1	0	15.7	1.7
	Abate et al. [35]	1998	30^c^					50	12
	Demissie et al. [40]	1998		12.9	0.6				
	Gebeyehu M et al. [23]	2001	203	18.2	0	31.6	0	19.5	0
	First National Drug resistance survey [7]	2003–2005	804		1.6		11.5		
	Desta K et al. [36]	2004–2005	297^b^	27.4	0				
	Abate D et al. [13]	2004–2008	376^c^			72.9	46.3		
	Asmamaw et al. [25]	2004–2005	173^b^	21.4	0.6				
	Agonafir M et al. [9]	2005–2006	114^c^	25	2.3	85.7	63.5	60.8	38.3
	Tessema B et al. [10]	2009	260	10.7	3.7	39.1	10.9	15.8	5.0
	Abebe G et al. [11]	2010–2011	136^b^	18.4	1.5				
	Esmael A et al. [37]	2010–2011	230	23.6	1.8	58.5	18.5	33.5	6.5
	Yimer et al. [26]	2012	112^b^	30.1	1.0				
	Seyoum et al. [8]	2011–2013	408^b^	23	1.1				
	Nigus et al. [12]	2012–2013	606^b^				15.3		
	Second National Drug resistance survey (7)	2014	1651		2.3		17.8		
	Mulisa G et al. [38]	2015	439^c^				33.2 %		

^a^ Of the study population, 106 TB cases were diagnosed

^b^ Only new cases

^c^ Previously treated and now presumptive for MDR-TB

## Discussions

Fifteen percent of the newly diagnosed, and fifty two percent of the previously treated TB cases were resistant to one or more of the first-line anti-TB drugs. MDR TB among new cases was 2.4 %, and 14.3 % among previously treated patients. Drug resistance was associated with previous history of TB treatment and urban residence. Resistances to any one or more of first-line anti-TB drugs and MDR-TB in the study population were high.

In this study, the 15 % resistance to any of the first-line anti-TB drugs among new TB cases is comparable to reports from Addis Ababa and Northern Ethiopia [10, 20]. However, it is higher than reports from other African countries [21–23] and yet lower than those from different parts of the country and elsewhere in Africa [8, 10, 11, 13, 24–29]. Moreover, the 52 % resistance rate to one or more first-line anti-TB drugs among previously treated TB cases in our study is high compared to previous reports from Ethiopia and others African counties [28–30]. However, it is lower than 54 % reported from Benin [31], 53.8 % from Somalia [32], 58.5 % from northern Ethiopia [10], the 71.4 and 72 % from Addis Ababa City [9, 13].

The difference in resistance rate to one or more first-line anti-TB drugs among new and previously treated TB patients across different study settings could be attributed to the variation in TB control programme performance, study population, sample size and study methods that were used across different geographical settings. For instance, the study subjects from Addis Ababa who had high rate of resistance stain [9, 13] were presumptive MDR-TB cases referred for MDR-TB investigation, while those recruited for the current study included any person 15 years age and above in the general population who had symptoms of TB and was at low risk of drug resistance.

Moreover, previous studies from Ethiopia and elsewhere in Africa were restricted to health service seekers at health facilities. Thus, study results from such segment of population may not indicate the real burden of the disease at community level as compared to the present population-based study. Likewise, the difference in burden of the resistance cases could be due to the time of the study; in the past the prevalence of resistant strains which might be a potential source of infection was not as high as they are today. For instance, in 2001, resistance to any first-line anti-TB drug among previously treated TB patients in Arsi Zone was 31.6 % [24]. However, after 14 years, it has reached 52.3 % in the study area. In general, the rate of primary and secondary resistance has been increasing in Ethiopia over the twenty years [7–12, 33–38]. Table 4 summarizes reports of previous studies on primary and secondary resistance compared to the current study in Ethiopia.

In the present study, previously treated TB cases were eight times more likely to have resistance to any of the first-line anti-TB drugs compared to new ones. The high level of anti-TB drug resistance among previously treated TB cases might have resulted from poor adherence and follow up or inadequate drug supply. This might result in selection of spontaneous mutation of *M. tuberculosis* strains [39]. Hence, the TB control programme has to explore reasons for such high secondary anti-TB resistances in the study area.

Moreover, resistance to any of the first-line anti-TB drugs was higher among urban residents when compared to their rural counterparts. This is in agreement with findings from a previous study in eastern Ethiopia which reported resistance of 81.7 % among urban dwellers 18.3 % among rural residents [8]. In fact, the association between MDR-TB and urban residence is not well established in the literature and may need further investigation. However, the most likely reason for the high prevalence of MDR-TB among urban residents might be because of higher HIV prevalence in urban settings and this may have increased the risk of MDR-TB infection [27, 40]. Moreover,, the high probability of exposure to anti-TB drugs from different sources in urban setting and the crowded living condition may also have contributed to increased transmission of resistant strains.

The 2.4 % prevalence of MDR-TB among the newly diagnosed TB cases in this study is very high compared to the 0 % reported in 2001 from the same area [24]. Nevertheless, it is similar to the 2.3 % reported by the 2014 National Drug Resistance and also by a study conducted in Addis Ababa [7, 9]. The relatively high rate of primary MDR-TB cases in the current population-based study could be due to the identification of the undiagnosed resistance cases in the community. Thus, these findings may imply the need for intensifying active TB case finding using community-based health extension workers and this could help in timely identification of undiagnosed resistant strains in Ethiopia [33].

The 14 % prevalence of MDR-TB among previously treated TB cases obtained in this study is lower than 18 % reported by the national surveillance [7], the 20.5 % of the global estimate [1] and other reports from elsewhere [30, 32, 41, 42]. However, it is high compared to the 0 % prevalence report from Arsi zone in 2001 [24]. Secondary resistance is mainly a result of poor Directly Observed Treatment Short course (DOTS) programme and should be as taken as a serious challenge in the TB control programme.

In this study, previously treated TB cases were more likely to have MDR-TB than the new ones. This is consistent with a meta-analysis in sub-Saharan Africa and a systematic review in Europe where a pooled risk of MDR-TB was higher among the previously treated TB cases as compared to the new ones [43, 44]. The overall high level of anti-TB drug resistance among re-treated TB cases compared to the newly diagnosed ones might be an indication of sub-optimal DOTS and DOTS-plus programme performance in Ethiopia.

The DOTS strategy aims to detect 70 % of infectious TB cases and achieve 85 % cure rate in order to interrupt transmission, reduce mortality and avert the emergence of drug resistance [45, 46]. However, reports from previous studies conducted in Ethiopia between 1984 and 2015 showed that the proportion of MDR-TB among new and previously treated TB cases varies from place to place and increased over time (Table 4). For instance, over 13 years, it increased from 0 to 2.4 % among new patients and from 0 to 14.3 % among previously treated ones in Arsi Zone [24]. Furthermore, according to previous reports, the fifteen years average TB case notification and cure rate of the study area were as low as 51.8 and 66.9 % [47, 48] respectively, very far from the 70 % global target of TB case notification and 85 % cure rate. Thus, the increasing trend in resistance to any TB drug and MDR-TB over time with the low TB case notification and cure rate in the study area may warrant alternative strategy to avert the emergence of drug resistance and strengthen TB control programme in Ethiopia.

Although DOTS- plus strategy is believed to be the best strategy to prevent emergence of MDR TB by accessing presumptive MDR-TB cases to diagnostic and appropriate treatment, MDR-TB patients are challenged by much more toxic and complicated treatment of longer duration which results in poor treatment outcome and emergence of XDR-TB. Therefore, effective implementation of DOTS and DOTS-plus strategies which are believed to be a corner stone in the prevention of the emergence and spread of MDR-TB and XDR-TB should be strengthen in the country. Thus, expanding MDR-TB diagnostic facilities and intensifying active case findings using health extension workers is urgent issues to be addressed in order to effectively control the increasing trend of drug resistance TB in Ethiopia [33].

To our knowledge, this is the first population-based study that analysed the prevalence of primary and secondary drug resistance and MDR-TB in Ethiopia. It might also be one of the very few studies conducted in poor resource setting. In fact, experienced and qualified laboratory technicians carried out the smear microscopy, sputum culture and DST to isolate *M. tuberculosis* and drug resistant strain. Thus we believe that the finding has highlighted the real burden of anti-TB drug resistance at community level. However the study is not without limitation. First, symptoms suggestive of TB were used as screening mechanism. The fact that chest X-ray was not used in the current study might have resulted in missed asymptomatic TB cases and underestimate the burden of the disease. Second, the current study excluded 20 contaminated sputum cultures which may have an impact on our result.

## Conclusions

The study has identified a high rate of primary and secondary resistance to any of the first-line anti-TB drugs and MDR-TB in the study area. The high rate of anti-TB drug resistance may have resulted from sub-optimal performance of DOTS programme in detecting infectious TB cases, and low cure rate in the study area. As anti-TB drug resistance is linked with pervious TB treatment, there is a need to strengthen DOTS and DOTS-Plus programme and expand MDR-TB diagnostic facilities so as to detect the cases in time and start appropriate treatment to prevent the spread of MDR-TB in Ethiopia.

## Abbreviations

AFB, Acid-Fast Bacillus; AOR, adjuster odd ratio; CI, confidence intervals; DOTS, directly observed short course treatment; DST, drug susceptibility tests; EMB, ethambutol; HEW, health extension worker; HIV, Human Immunodeficiency Virus; INH, isoniazid; IUATLD, International Union against Tuberculosis and Gung Disease; LJ, Löwenstein-Jensen; MDR-TB, multi- drug-resistant TB; RIF, rifampicin; STM, streptomycin; TB, tuberculosis; WHO, World Health Organization; XDR-TB, extensively drug-resistant TB

## Additional files

Additional file 1: Dataset supporting conclusions of the study on primary and secondary MDR-TB of Hitossa District of Arsi Zone, 2016, submitted to BMC public health using STATA software. (DTA 16 kb) Additional file 2: Dataset supporting conclusions of the study on primary and secondary MDR-TB of Hitossa District of Arsi Zone, 2016, submitted to BMC public health using excel software. (CSV 22 kb)
